# Laparoscopic cholecystectomy for giant gall stone: Report of two cases

**DOI:** 10.1016/j.ijscr.2020.01.055

**Published:** 2020-02-06

**Authors:** P.O. Igwe, O.N. Diri

**Affiliations:** Department of Surgery, University of Port Harcourt Teaching Hospital Alakahia, Rivers State, Nigeria

**Keywords:** Giant gall stone, Laparoscopic cholecystectomy

## Abstract

**Background:**

Giant Gallbladder calculus is an uncommon finding among Africans. This condition has paucity of reports in literature. Laparoscopic approach is even a rarer modality of treatment.

**Aim:**

This is to report two cases of giant gall stone treated laparoscopically.

**Case Report:**

A 32 year old female presented with a history of right hypochondrial pain, on examination, blood pressure was 110/70 mmHg, pulse was 76 beats per minute, and tender right hypochondrum. Abdominal ultrasound showed calculus in gall bladder working diagnosis was Acute Cholecystitis secondary to Cholelithiasis.

She had laparoscopic cholecystectomy. The findings were: Giant gallbladder calculus measuring 8.2 cm by 7.5 cm in diameter.

The second patient was a 62 year old woman who presented with right hypochonrial pain, on examination, Murphy’s and Moynyham modified Murphy’s were positive. Abdominal and pelvic scan showed multiple gallbladder calculi. She had laparoscopic cholecystectomy and findings were multiple calculi with one measuring 8 cm by 6 cm in widest diameters. Both patients’ follow-up in outpatient clinic were uneventful.

**Conclusion:**

Giant gall stone is a rare condition. It is not an absolute indication for open cholecystectomy. Laparoscopy can be a very useful method of treatment in experienced hands.

## Highlights

• The size of the gall stone has been frequently used as an index for tendency of a conversion from Laparoscopic to open cholecystectomy. We used laparoscopic approach to treat a giant gall stone successfully.
• The dexterity and experience of the surgeon is what determines how safe choice of approach is irrespective of the size of the gall bladder.
• We widened the epigastric port to retrieve the stone in first case but in the second case we widened the umbilical port.

## 1 Introduction

Gall bladder stone is a common condition in Africa. Giant gall bladder stone is very rare [1,2]. Giant Gall bladder stone can be defined as stones larger than 5 cm in its widest diameter or weighing above 70 g [3,4]. Some literature referred to the condition as an indication for open cholecystectomy [[4], [5], [6]] but we have a different opinion and hence the report of two cases managed through laparoscopic cholecystectomy. This work has been reported in line with the SCARE criteria [7].

Case Report:

A 32 year old female presented with a history of right hypochondrial pain, On examination, blood pressure was 110/70 mmHg, pulse was 76 beats per minute, and tender right hypochondrum. Abdominal ultrasound showed calculus in gall bladder. Working diagnosis was Acute Cholecystitis secondary to Cholelithiasis.

**Fig. 1:**
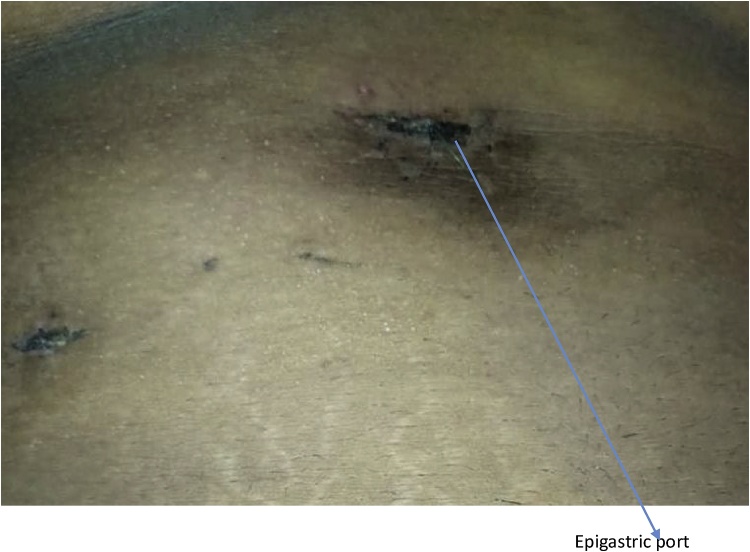
Post-operative day 3 of widened epigastric port.

**Fig. 2:**
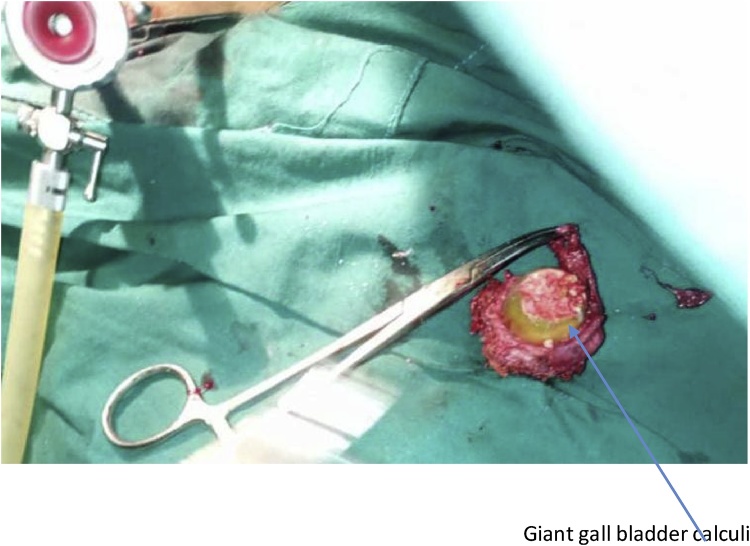
Giant gallstone.

**Fig. 3:**
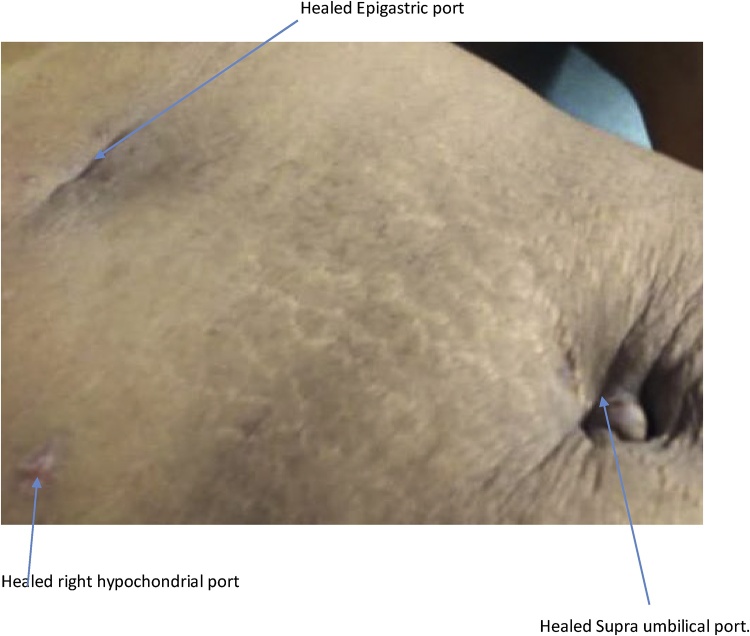
Healed laparoscopic scar on 7th day post-operative.

She had laparoscopic cholecystectomy. Fig. 1 at post-operative day 3. The findings were: Giant gall bladder calculus measuring 8.2 cm by 7.5 cm in diameter (Fig. 2). Her 7th day Post-operative wound showed normal healing (Fig. 3). Histology report showed acute cholecystitis from cholelithiasis.

**Fig. 4:**
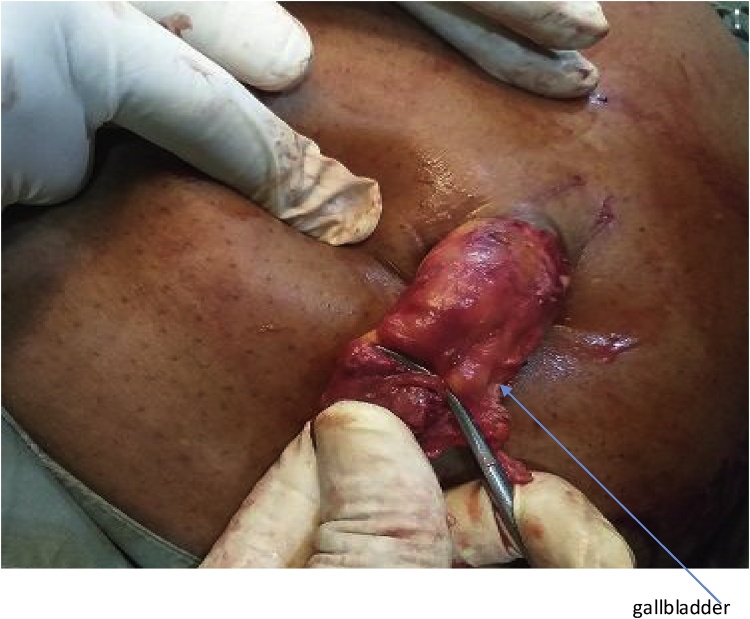
Gall bladder retrieval from the Supra-umbilical port.

**Fig. 5:**
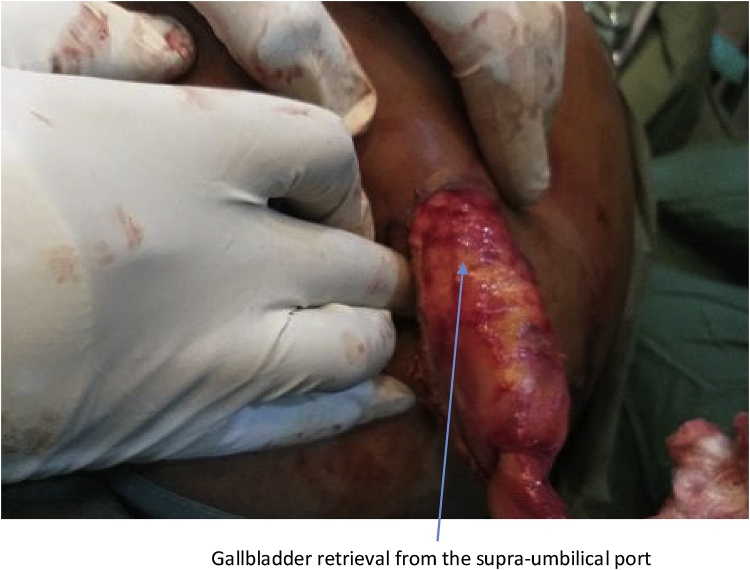
Gallbladder retrieval from the Supra-umbilical port.

**Fig. 6:**
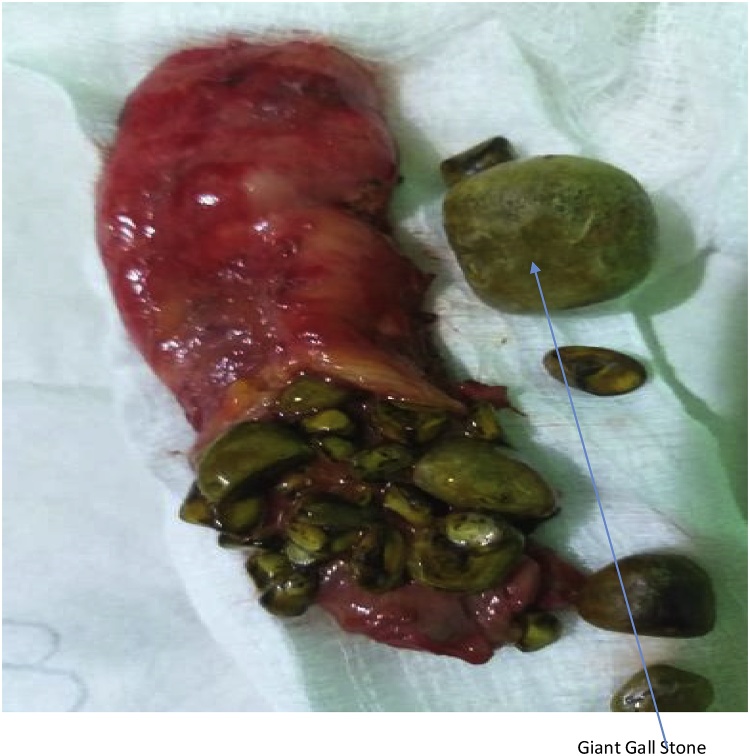
Gallbladder with multiple calculi and the Giant gall stone.

**Fig. 7:**
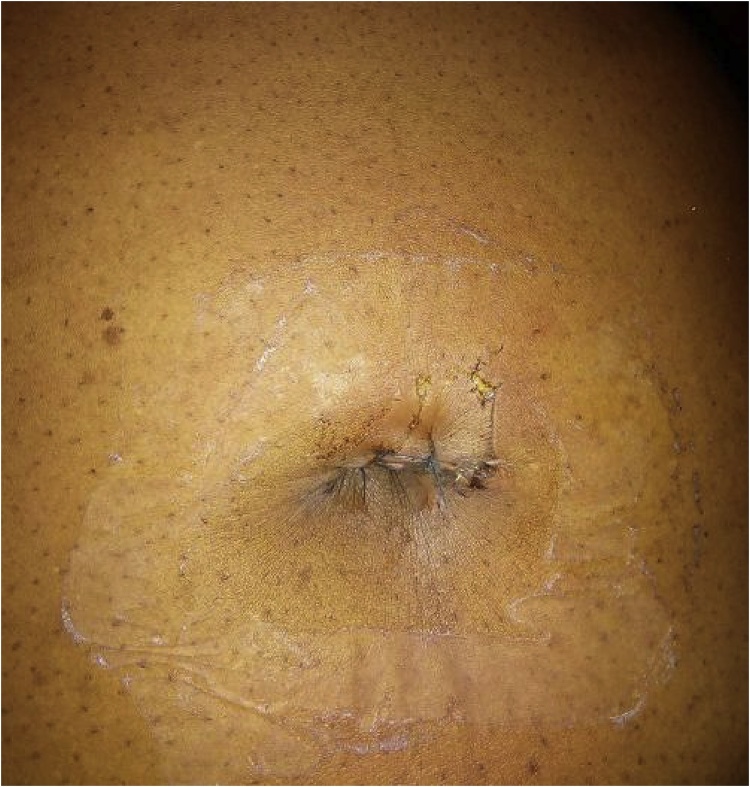
Widened Supra-umbilical port at post-operative day three.

The second patient was a 62 year old woman known diabetic and hypertensive who presented with right hypochondrial pain of three years duration, no fever, jaundice and itching. On examination, Murphy’s and Moynyham modified Murphy’s were positive. Bowel sounds were present and normoactive. Digital rectal examination was unremarkable. Abdominal and pelvic scan showed multiple gall bladder calcuuli. She had laparoscopic cholecystectomy and retrieval of gallbladder was done through the supra-umbilical port (Fig. 4, Fig. 5). Intra-operative findings were multiple calculi with one of the stones measuring 8 cm by 6 cm in widest diameters (Fig. 6). Her 3rd day Post-operative wound scar show normal healing (Fig. 7). Her follow-up in outpatient clinic was uneventful.

## 2 Discussion

A stone formed in gall bladder is termed Gall stone disease. Gall stone disease is a very common clinical condition. This condition is more common in western world than Asia and Africa with females being more commonly affected than men [1]. Giant gall stones reported in literature are quite rare. It may be defined as those greater than 5 cm in their widest diameter [3,4]. It is even rarer to see giant gall stone treated laparoscopically. In fact giant gall stone is reported to be an indication for conversion from laparoscopic to open cholecystectomy [[4], [5], [6]].

The aetiological factors leading to gall stone formation could be multi-factorial such as infection, Metabolic and stasis or combination of these factors. one of the most common medical complications leading to surgical intervention is attributable to gall stone disease especially in sickle cell disease patients, diabetics, patients who undergone weight reduction surgeries. It could be asymptomatic or sometimes develop to a symptomatic stage [8] as in our patients.

Acute cholecystitis due to cholelithiasis can be diagnosed clinically. Radiological investigations play a key role prior to surgery. Besides confirming gall stone, it could estimate the size and some complications that may be associated. Ultrasound scan is very sensitive in diagnosing gall stone, it can accurately assess the size of a gallstone and should be done routinely [9,10]. Abdominal ultrasound appears to be better than computerized axial tomographic scan in diagnosis of cholelithiasis. In our two cases, scan reported presence of calculi in gall bladder with their sizes but computerized axial tomographic scan was unable to diagnose stones in second patient where it was requested, and in fact reported it as acalculous cholecystitis. This may be due to the nature of the stones, as cholesterol stones are rarely radio-opaque, hence made it difficult to be seen on CT. Although the thickened walls of the gall bladder was reported on CT.

The risk of biliary tract injuries represent the potentially life-threatening complication of laparoscopic cholecystectomy. The risk increases in case of inflammation or anatomical anomaly. Some authors have evaluated the role of preoperative magnetic resonance cholangiopancreatography (MRCP). Preoperative MRCP with a thorough description of biliary tract anatomy might allow safer laparoscopic cholecystectomy procedure [11,12].

The gall bladder may contain a single large stone as in our first index case or many smaller ones in addition. It may also contain both giant with multiple stones as we saw in our second index patient. This is yet to be reported in literature to the best of our knowledge and literature search. Gallstones with a diameter of over five cm are very rare. Giant gall bladder stones, one measuring four inch in circumference and another six and a half inch long and six inch thick have been reported [4,5]. Also gallstone of size 74 × 54 mm and weighed 72 g and composed of bilirubin and cholesterol with traces of organic matter has been reported by Satish Dallal et al. [4].

Patients with large gallstones may be more suited to admission to an inpatient unit as they have a higher risk of conversion to open. The gold standard treatment for symptomatic gall stones currently is Laparoscopic cholecystectomy. Large gallstones may pose technical difficulties when performing a laparoscopic cholecystectomy.

laparoscopic cholecystectomy have been reported to be a difficult procedure in presence of giant gall stone due to more severe inflammation and thickening of the gallbladder wall. The large gallstone would make it difficult to grasp the gallbladder with the laparoscopic instruments and expose the anatomy of Calot’s triangle. However this is possible and virtually depends on the skill and experience of the laparoscopic surgeon. In The two cases we did we were able to dissect the gall bladder out. Difficulty with the retrieval of such a large gallstone has also been reported, however we widened the epigastric port to retrieve the stone in first case but in the second case we widened the umbilical port and exchange the optical with epigastric port. This can create mirror image which the surgeon has to anticipate during retrieval while using this approach as we did. Better wound scar was relatively noted with umbilical retrieval. The histology result showed acute cholecystitis in the first case and chronic cholecystitis in second case. Port site metastasis was also our concern but no evidence of malignancy was reported on histology. Strictly speaking, giant gall bladder stone does not pose absolute indications for conversion to open cholecystectomy as seen in our index cases. Therefore, Laparoscopic cholecystectomy is still the procedure of choice in such cases instead of Open cholecystectomy unlike what is reported by some Author [4]. This especially when the surgeon is experienced and skillful, size may not be an indication for conversion to open cholecystectomy.

The size of the gall stone has been frequently used as an index for tendency of a conversion from Laparoscopic to open cholecystectomy. Large or giant gallstones would result in more severe inflammation and thickening of the gallbladder wall. Giant gallstone is said to make gall bladder difficult to be grasped with the laparoscopic instruments in other to expose the anatomy of the Calot's triangle [4]. We used laparoscopic approach to treat a giant gall stone successfully. In both cases we did not convert to open cholecystectomy. The dexterity and experience of the surgeon is what determines how safe choice of approach is, irrespective of the size of the gall bladder. In conditions where the Calot's triangle is difficult to be exposed because of adhesion or inability to grasp the gallbladder open is considered to be safer [[13], [14], [15]]. this is unlike in our cases where the Calot’s triangle was difficult to be dissected, the use or change of energy source may be better decision and also the type of grasper used. To the best of our knowledge, these cases were the first report in literature about a patient with such a very rare giant gallstone undergoing Laparoscopic cholecystectomy where the retrieval was done via the umbilical port successfully. No retrieval bag was used No evidence of ports site infection and metastasis, in a diabetic patient.

During cholecystectomy, even in open or laparoscopic approach, surgeons should not allow the gallbladder to break or rupture. Firstly, to forestall spread stones or spillage of bile in abdomen with high risk of abdominal infections. Secondly, there can be a risk of unknown cancer or metastasis in gallbladder [16,17], so surgeons should protect the extraction of gallbladder with "endobag". However some Authors have reported merits and demerits of use of endobag [18,19]. In our first case an improvised endobag (sterile glove) was fashioned and used to retrieve the gall bladder part, following rupture due to traction-induced transverse arrest at the epigastric port site.

The actual purpose for conversion to open is inability to expose the anatomy [13]. Although is reported in literature that Giant gallstones within the gallbladder can necessitate a conversion to open surgery by two ways [[20], [21], [22]]. Firstly, Giant stones in the gallbladder may contribute to inflammation and thickening of the gallbladder wall. Secondly, a large gallstone can make it difficult to grasp the gallbladder with laparoscopic instruments and expose the anatomy to be dissected. It is difficult to ascertain when a gallstone should be considered ‘large’ and more studies are required. The location of the gallstone may also be an important predictive factor. Prior to surgery, the use of ultrasound measurements of the thickness of gallbladder wall has been shown to correlate with conversion to open surgery in bivariate analyses [14,23,24]. This area still needs further studies to authenticate this claim. More studies are required to ascertain what measurements should be used to define a gallstone as ‘large’ in relation to an increased risk of conversion to open.

## 3 Conclusion

Giant gall stone is rare, it is not an absolute indication for open cholecystectomy. Laparoscopy can be a very useful method of treatment in experienced hands.

## Conflicts of interest

No conflict of interest.

## Sources of funding

No source of funding.

## Ethical approval

Exemption of ethical approval was given because no identifiable patient’s parts were seen.

## Consent

Written and signed consent to publish as case report was obtained from both patients.

## Author contribution

PO Igwe; case design and write up.

ON Diri; proof read and approve with corrections.

## Registration of research studies

Case series.

## Guarantor

PO Igwe.

## Provenance and peer review

Not commissioned, externally peer-reviewed.
